# Alterations in the properties of the cell membrane due to glycosphingolipid accumulation in a model of Gaucher disease

**DOI:** 10.1038/s41598-017-18405-8

**Published:** 2018-01-09

**Authors:** Gyula Batta, Lilla Soltész, Tamás Kovács, Tamás Bozó, Zoltán Mészár, Miklós Kellermayer, János Szöllősi, Peter Nagy

**Affiliations:** 1 Department of Biophysics and Cell Biology, Faculty of Medicine, University of Debrecen, Debrecen, Hungary; 2 Department of Genetics and Applied Microbiology, Faculty of Science and Technology, University of Debrecen, Debrecen, Hungary; 3 Department of Biophysics and Radiation Biology, Semmelweis University, Budapest, Hungary; 4 Department of Anatomy, Histology and Embryology, Faculty of Medicine, University of Debrecen, Debrecen, Hungary; 5 MTA-DE Cell Biology and Signaling Research Group, Faculty of Medicine, University of Debrecen, Debrecen, Hungary

## Abstract

Gaucher disease is a lysosomal storage disease characterized by the malfunction of glucocerebrosidase resulting in the accumulation of glucosylceramide and other sphingolipids in certain cells. Although the disease symptoms are usually attributed to the storage of undigested substrate in lysosomes, here we show that glycosphingolipids accumulating in the plasma membrane cause profound changes in the properties of the membrane. The fluidity of the sphingolipid-enriched membrane decreased accompanied by the enlargement of raft-like ordered membrane domains. The mobility of non-raft proteins and lipids was severely restricted, while raft-resident components were only mildly affected. The rate of endocytosis of transferrin receptor, a non-raft protein, was significantly retarded in Gaucher cells, while the endocytosis of the raft-associated GM1 ganglioside was unaffected. Interferon-γ-induced STAT1 phosphorylation was also significantly inhibited in Gaucher cells. Atomic force microscopy revealed that sphingolipid accumulation was associated with a more compliant membrane capable of producing an increased number of nanotubes. The results imply that glycosphingolipid accumulation in the plasma membrane has significant effects on membrane properties, which may be important in the pathogenesis of Gaucher disease.

## Introduction

The plasma membrane constitutes an interface between the cell and its surroundings, and it is the site of numerous transmembrane signaling and membrane trafficking events. These functions depend on a balance between membrane rigidity and flexibility, which is, in turn, influenced by the complex composition of the membrane and its interactions with the extracellular matrix and the cytoskeleton^1^. The cell membrane contains hundreds of different types of lipids^1^. Alterations in the representation of certain lipids are expected to have significant effects on membrane properties providing explanation for certain disease symptoms and therapeutic approaches^2^.

Gaucher disease is the most common lysosomal storage disorder, which is inherited in an autosomal recessive way. Glucosylceramide and other sphingolipids accumulate in swollen lysosomes of affected cells due to the malfunction of glucocerebrosidase (GBA1), a lysosomal enzyme that normally degrades them^3^. Based on the type and severity of symptoms Gaucher disease is classified into neuronopathic (types II and III) and non-neuronopathic forms (type I), in which neurons and macrophages, respectively, are the primarily affected cell types. Accumulation of sphingolipids leads to cytokine secretion and the accumulation of glucosylsphingosine, a neurotoxin, leading to inflammation and neurodegeneration^4^. A multitude of mutations in GBA1 have been identified, and although some of them are associated with certain types of Gaucher disease (e.g. the N370S and L444P alleles predominantly lead to type I and II diseases, respectively), disease symptoms weakly correlate with the type of mutation, residual enzyme activity and the amount of stored lipids^5^. Therefore, other mechanisms have been implicated in the development of the symptoms including the activation of ER stress pathways, which can also shed light on the association of GBA1 mutations and Parkinsonism^6^.

Since all membrane compartments are interconnected with each other by vesicular transport and lipid transport proteins, it is expected that the accumulation of glucosylceramide and sphingolipids is not limited to lysosomes. Indeed, increased concentration of these lipids have been observed in the plasma membrane in *in vitro* and *in vivo* models^7–9^. Such alterations in the composition of the plasma membrane are expected to change its biophysical and cell biological properties.

The nature and even the existence of lipid rafts are fiercely contested. They are generally believed to be cholesterol- and sphingolipid-enriched microdomains with submicroscopic size thought to correspond to liquid-ordered domains observed in artificial membranes^10–12^. However, there are striking differences between the stability and size of liquid-ordered domains in model membranes and lipid rafts in living cells, raising doubts about the very existence of rafts^13,14^. According to a reasonable compromise between the conflicting views lipid rafts may be envisaged as submicroscopic, highly dynamic microdomains enriched in cholesterol, sphingolipids and certain proteins whose existence is not primarily caused by lipid partitioning, but by protein-lipid interactions, membrane trafficking and interactions of the membrane with the cytoskeleton^15,16^.

Here we used an *in vitro* model of Gaucher disease in which the activity of glucocerebrosidase was inhibited by conduritol B epoxide in THP-1 monocyte-derived macrophages^9^. Our aim was to investigate the effect of sphingolipids accumulating in the plasma membrane on the biophysical and cell biological properties of the membrane. The results show that sphingolipid accumulation has more profound effects on non-raft components than on raft components concerning lateral mobility and rate of endocytosis. A model is proposed in which the coalescence of enlarged raft-like domains accounts for the alterations in the properties of the plasma membrane.

## Results

### The change in membrane composition in Gaucher-type cells significantly alters the diffusion properties of non-raft molecules in the cell membrane

**Figure 1:**
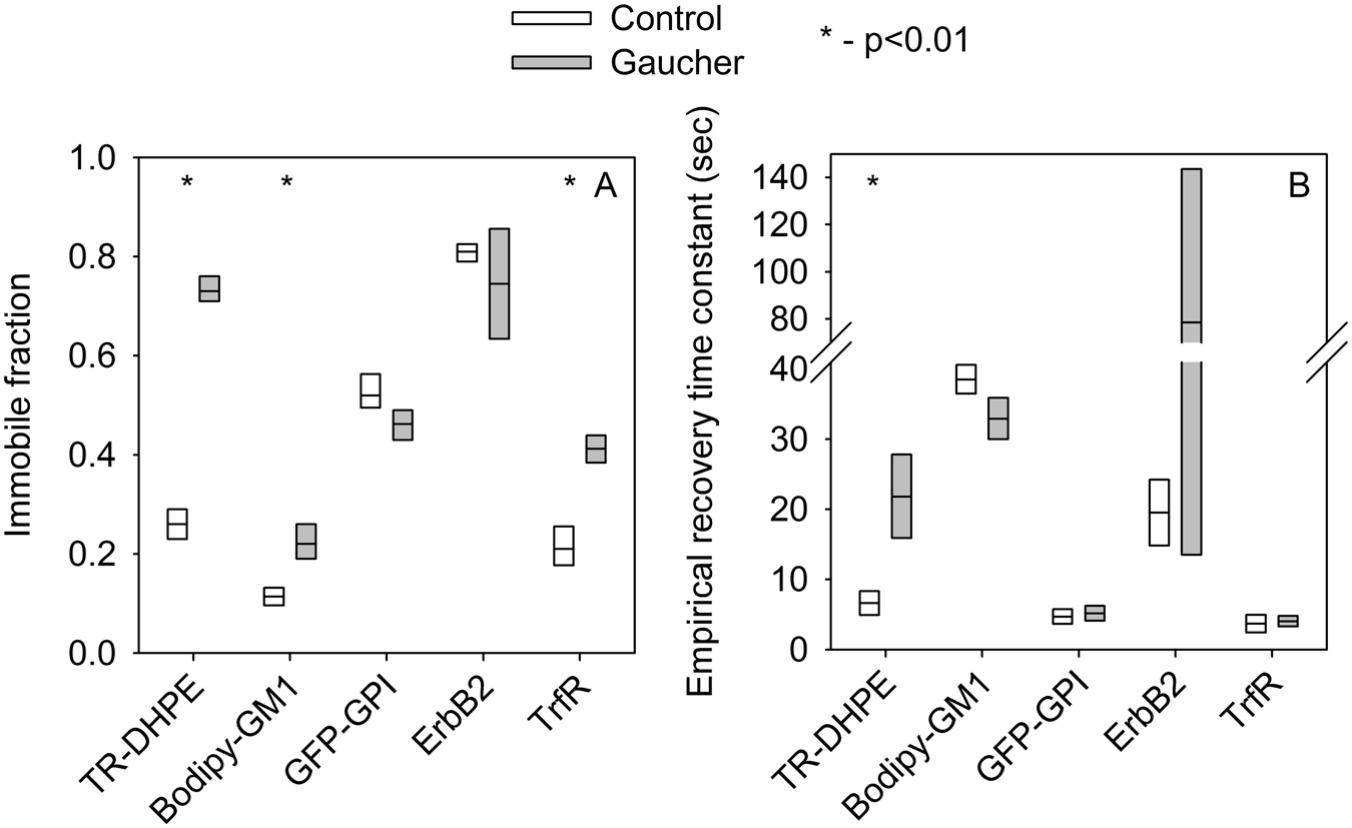
Measurement of the lateral diffusion of membrane proteins and lipids by FRAP. Fluorescent lipid analogs were incorporated into the membrane, or membrane proteins coupled to a fluorescent protein variant were transfected to control or Gaucher-type, sphingolipid-enriched macrophages. The immobile fraction (**A**) and the rate of lateral diffusion, characterized by the empirical recovery time constant (**B**), were determined by fitting equation 2 to the averaged, double-normalized recovery curves shown in Fig. S4. The line within the box indicates the fitted immobile fraction and recovery time constants, whereas the boundaries of the boxes correspond to the 95% confidence bounds. The graphs are based on ten FRAP experiments from three independent labelings or transfections, in which a single region was bleached in a single cell. Statistical significance was estimated by the extent overlap between the confidence intervals of the fitted parameters.

The mobility of different membrane components is a peculiar feature of the plasma membrane, which determines its functional properties. In order to identify the changes in these characteristics in Gaucher model cells, we performed fluorescence recovery after photobleaching (FRAP) measurements to determine the immobile fractions and mobility of lipids and membrane proteins. Since it has been shown previously that the amount of sphingolipid species increases by a factor of 2–10 in Gaucher-type macrophages, these cells can be used for characterizing the effect of sphingolipid accumulation on the biophysical properties of the cell membrane^7–9^. Treatment with conduritol B epoxide (CBE), an inhibitor of glucocerebrosidase (lysosomal acid β-glucosidase), was used to induce the Gaucher phenotype *in vitro* ^7,9^. This chemically induced model of the disease has been used extensively for the characterization of disease symptoms and cellular changes associated with sphingolipid accumulation, and the CBE-induced and knock-out models of Gaucher disease show remarkably similarities with regard to pathological features and gene expression profile^17,18^. Although CBE may inhibit the activity of non-lysosomal β-glucosidase (GBA2) as well^19^, this effect also results in glycosphingolipid accumulation supporting the appropriateness of the model system for studying the consequences of glycosphingolipid accumulation^20^. CBE treatment did not modify the viability of cells (Fig. S1). Membrane lipid analogues Texas Red dihexadecanoyl-phosphatidylethanol amine (TR-DHPE) and Bodipy FL-labeled GM1 are preferentially found outside and inside lipid rafts, respectively^21,22^. Here, the non-overlapping labeling of the plasma membrane by the lipid analogs was confirmed by confocal microscopy (Fig. S2). Besides the lipid analogues, we also analyzed three proteins selected for their different relationships to lipid rafts. GFP-GPI (glycosylphosphatidylinositol-anchored green fluorescent protein) and ErbB2 are strongly and weakly associated with lipid rafts, respectively, while transferrin receptor is outside this microdomain^23–25^. Representative recovery curves of individual cells and their fitting are shown in Fig. S3, while supplementary movie 1 demonstrates the FRAP image sequence. Recovery curves were averaged and these mean recovery curves were fitted to reveal the immobile fractions and the recovery time constants (Fig. S4). The immobile fraction of the non-raft lipid TR-DHPE was 3-times larger in Gaucher type model cells compared to the control, while the immobile fraction was less substantially modified for the raft-associated Bodipy FL-GM1 (Fig. 1A, Fig. S4). A similar difference was revealed for the raft and non-raft protein components as well with the immobile fraction of transferrin receptor two-times larger in sphingolipid-enriched cells, while immobile fractions of raft-resident GFP-GPI and ErbB2 were not significantly affected. A similar tendency was observed for the rate of lateral diffusion of lipids. While the diffusion rate characterized by the recovery time constant was significantly lower for TR-DHPE in Gaucher-type cells, the recovery rate of Bodipy FL-GM1 did not change significantly. The diffusion rate of proteins was not modified significantly upon sphingolipid accumulation (Fig. 1B, Fig. S4). In conclusion, protein and lipid components associated with the non-raft phase of the cell membrane are much more sensitive to the glycosphingolipid accumulation characteristic of Gaucher-type model cells compared to raft constituents.

### The cell membrane of Gaucher model cells is characterized by lower fluidity and hydration

**Figure 2:**
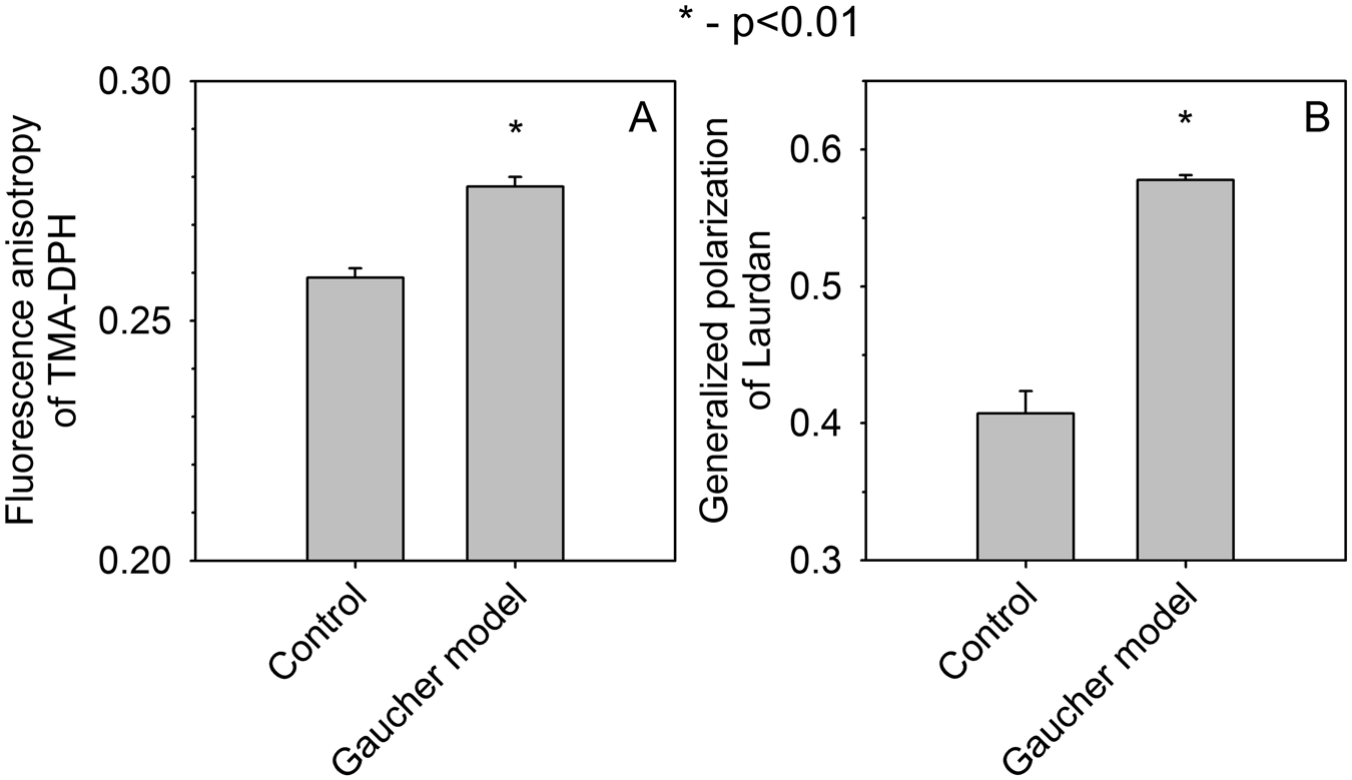
Measurement of membrane fluidity and hydration. Control and sphingolipid-enriched Gaucher-type macrophages were labeled with TMA-DPH or Laurdan and the fluorescence anisotropy of TMA-DPH (**A**) as well as the generalized polarization of Laurdan (**B**) were measured by fluorometry. The error bars display the standard error of the mean (n = 10 from four independent experiments). Control and Gaucher-type cells were compared by two-way ANOVA followed by Tukey’s HSD test. Asterisks indicate significant differences between the control and Gaucher-type cells.

Since lateral mobility of membrane proteins and lipids depends on the appropriate fluidity of the cell membrane, we set out to compare Gaucher cells and their control counterparts regarding the fluidity and hydration of their membrane. Cells were labeled with 4′-(trimethylammonio)-diphenylhexatriene (TMA-DPH), and its fluorescence anisotropy, reporting on the rotational freedom of the indicator in the nanosecond time range^26^, was measured by fluorometry. The results imply that the rotational mobility of TMA-DPH is more restricted in the membrane of Gaucher-type model cells compared to the control suggesting that glycosphingolipid accumulation in Gaucher cells increases the microviscosity of the cell membrane (Fig. 2A). Spectral shifts in the emission spectrum of Laurdan, expressed by its generalized polarization, characterize membrane order and hydration^27^. The fact that the generalized polarization of Laurdan is significantly increased in CBE-treated cells compared to control samples implies that the membrane of Gaucher model cells is less hydrated and more ordered than that of control cells (Fig. 2B). Two-photon microscopic images recorded from the middle plane of cells confirmed that most of the fluorescence of TMA-DPH and Laurdan originated from the cell membrane under the experimental conditions used for imaging (next section) and fluorometry (Fig. S5).

### Lipid rafts occupy a larger fraction of the cell membrane leading to their coalescence in Gaucher cells

**Figure 3:**
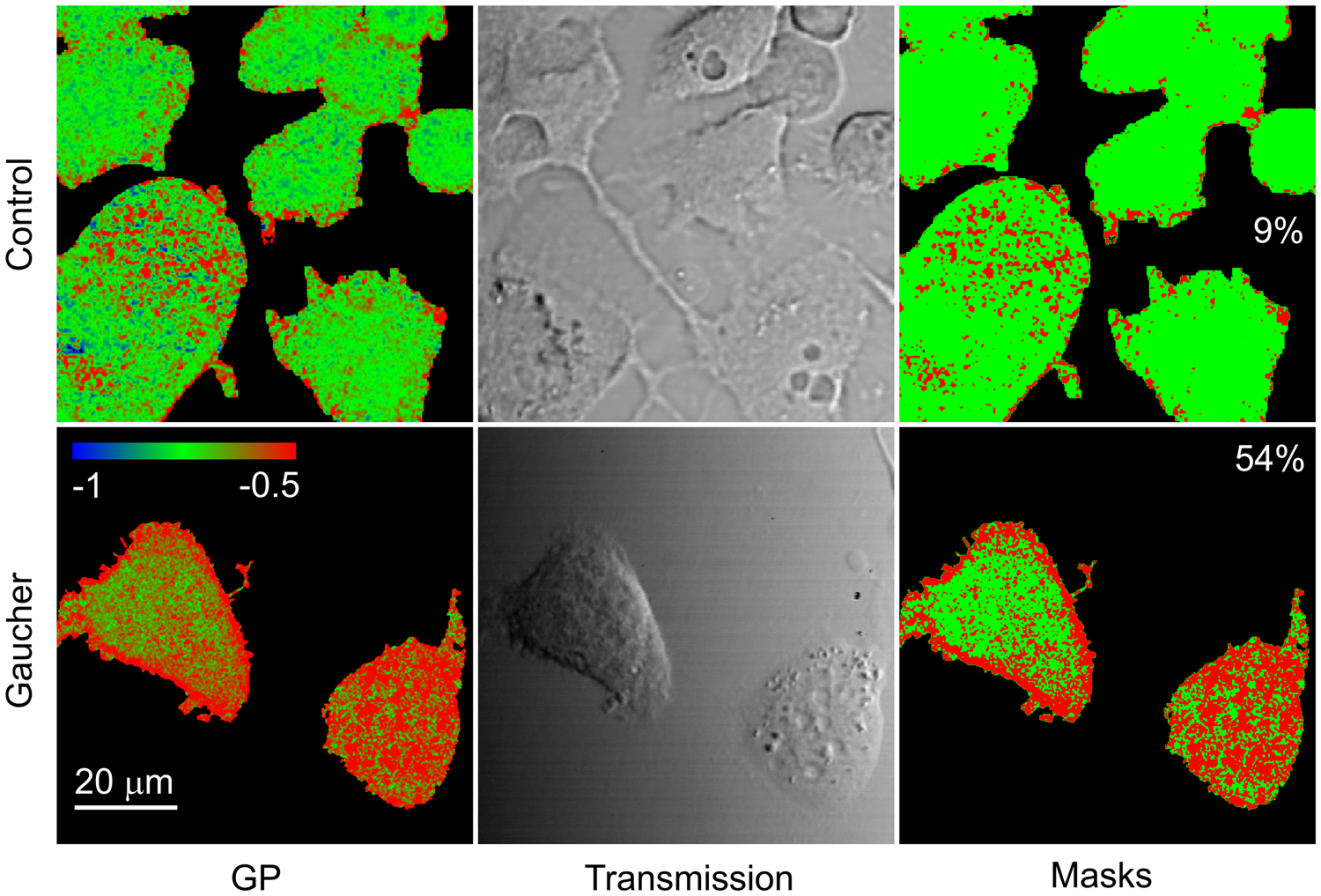
Two-photon microscopic measurement of the generalized polarization of Laurdan. Control and sphingolipid-enriched Gaucher-type macrophages were labeled by Laurdan and the generalized polarization (GP) of the indicator, characterizing membrane hydration, was measured by two-photon microscopy. Images were recorded in a plane corresponding to the flat plasma membrane adjacent to the coverslip. The generalized polarization of the indicator incorporated into the plasma membrane is shown on the left on a color scale ranging between -1 and −0.5. Hydrated and less hydrated membrane domains are shown in green and red, respectively. The generalized polarization values shown in these images are not comparable to the ones in Fig. 2, since generalized polarization of the indicator is modified by the sensitivity of detectors recording the blue and red spectral range of Laurdan emission, which was different for the fluorometric and microscopic experiments presented in Figs 2 and 3, respectively. The corresponding transmission images showing the morphology of cells are displayed in the middle. The images on the left were segmented into two masks corresponding to high and low values of generalized polarization shown in red and green, respectively. The same threshold was applied for both images, and it was chosen by visual inspection. The percentages displayed on the masks are the fractional areas of the high-GP mask.

The membrane localization of Laurdan allows us to map the distribution of membrane order and hydration. Besides confirming the fluorometric results (Fig. 2), two-photon microscopic images revealed that the cell membrane of Gaucher type cells contains an increased ratio of highly-ordered membrane, characterized by high generalized polarization, compared to control samples (Fig. 3). While the majority of the membrane of control cells was occupied by non-raft microdomains of low order, the overall area of lipid rafts in Gaucher-type cells was overwhelming. Quantitative analysis revealed that the fractional area of membrane areas with a high generalized polarization increased from 13 ± 2% in control cells to 48 ± 5% in Gaucher-type cells (mean ± SEM, n = 9, p < 0.05). Gross alterations in the amount of membrane could lead to changes in membrane load or membrane tension^28,29^, which could also modify membrane hydration. Since such gross alterations in membrane load would be reflected in an altered cell shape, we determined how spread-out control and Gaucher-type cells were by determining their height normalized by the area of their membrane adjacent to the coverslip. Since the normalized height of control and Gaucher-type cells was identical within experimental error (Fig. S6), we concluded that gross alterations in cell shape could not account for the observed changes in membrane hydration. Thus, lipid rafts are likely to have coalesced, at the resolution limit of two-photon microscopy, leading to the formation of a continuous, percolating raft-like phase.

### Atomic force microscopy reveals alterations in the formation of membrane tethers in Gaucher-type macrophages

**Figure 4:**
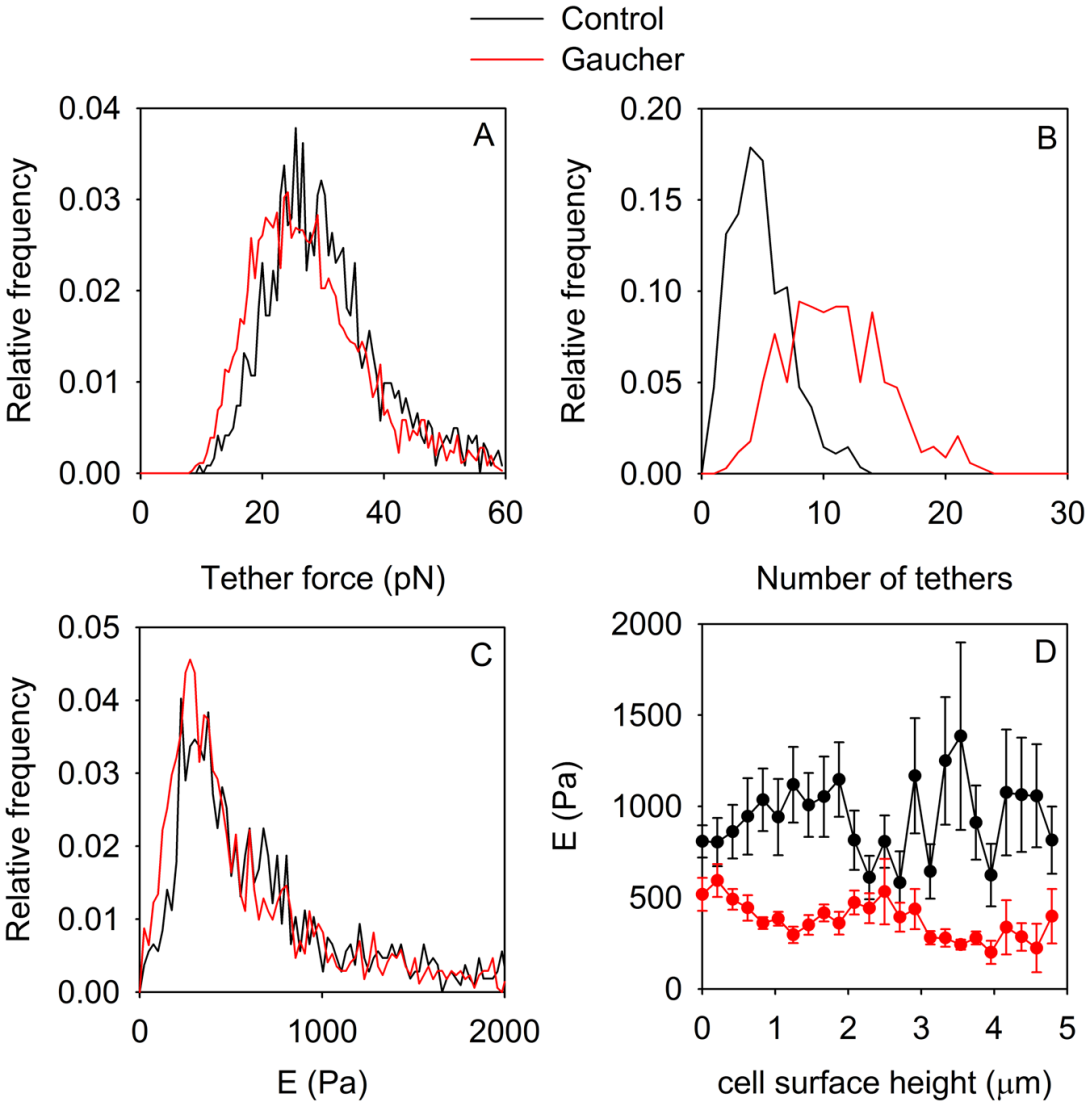
Measurement of membrane tether formation and elastic properties using atomic force microscopy. (**A**,**B)** Membrane tether formation was analyzed on the force vs. separation curves corresponding to the retraction phase by atomic force microscopy. Tether force was determined as the abrupt change in force upon detachment of a tether from the cantilever and the distribution of tether forces in control and Gaucher-type cells was calculated (**A**). Due to the large number of elements (n = 1319 and n = 3741 for the control and Gaucher-type cells, respectively) the histogram means are significantly different from each other (p < 0.001). Such step-like changes in the force curve were enumerated and the distributions of their numbers for control and Gaucher-type cells are displayed (**B**). The means of the number of membrane tethers are significantly different from each other (p < 0.01). (**C**,**D)** The elastic modulus was determined in the extension phase of the force vs. separation curve, and its histogram in control and Gaucher-type cells is shown in (**C**). The height of the AFM cantilever above the cell was divided into 25 bins and the mean of Young’s modulus calculated in each bin was plotted as a function of the height (**D**).

Atomic force microscopy (AFM) is a versatile technique suitable for determining topographical and nanomechanical properties of the membrane^28,30,31^. Force-volume maps were recorded in 16×16 matrices overlaid on individual cells. When the AFM cantilever is retracted from the cell, membrane tethers, corresponding to nanotubes, are formed between the membrane and the AFM tip. These tethers are believed to characterize the stiffness of the membrane and its interaction with the underlying cytoskeleton^32,33^. Stepwise changes in the retraction force spectrum correspond to extension and rupture of individual tethers allowing us to enumerate them and to measure the single-tether force (Fig. S7). The median force was 30 pN and 27 pN in control and Gaucher-type macrophages, respectively (Fig. 4A). The 10% decrease in the tether force is statistically significant due to the large number of elements in both samples. Thus, the Gaucher-type membrane tethers require less force to be extended, hence their surface tension or bending rigidity is smaller^34^. The Gaucher phenotype led to a doubling in the number of tethers, with the mean tether number increasing from 4.8 ± 2.4 to 11.1 ± 4.3 (mean ± SD, Fig. 4B). We concluded that the cell membrane of Gaucher-type macrophages was more prone to the formation of membrane nanotubes than that of control cells.

In order to determine the elasticity of the cell and the plasma membrane we fitted a Hertzian model to the indentation part of the force curves to obtain Young’s modulus. Although the histogram of the elastic modulus showed a hardly detectable shift to lower values in Gaucher-type cells, two measures of central tendency clearly implied that the cell became significantly more compliant upon accumulation of glycosphingolipids (Fig. 4C, median elastic modulus: 614 Pa and 459 Pa in control and Gaucher-type cells, respectively; mean elastic modulus: 1031 Pa and 795 Pa in control and Gaucher cells, respectively; p < 0.01 according to Mann-Whitney test). Since the elastic modulus might be different above the nucleus due to differences in the stiffness of the nucleus and the cytoplasm, and due to artifacts, we determined the average of Young’s modulus as a function of the local topographical height. The data convincingly shows that Gaucher-type cells have a significantly lower elastic modulus regardless of cellular height (Fig. 4D).

### Endocytosis of transferrin and subunit B of cholera toxin is differentially affected by sphingolipid accumulation in the plasma membrane

**Figure 5:**
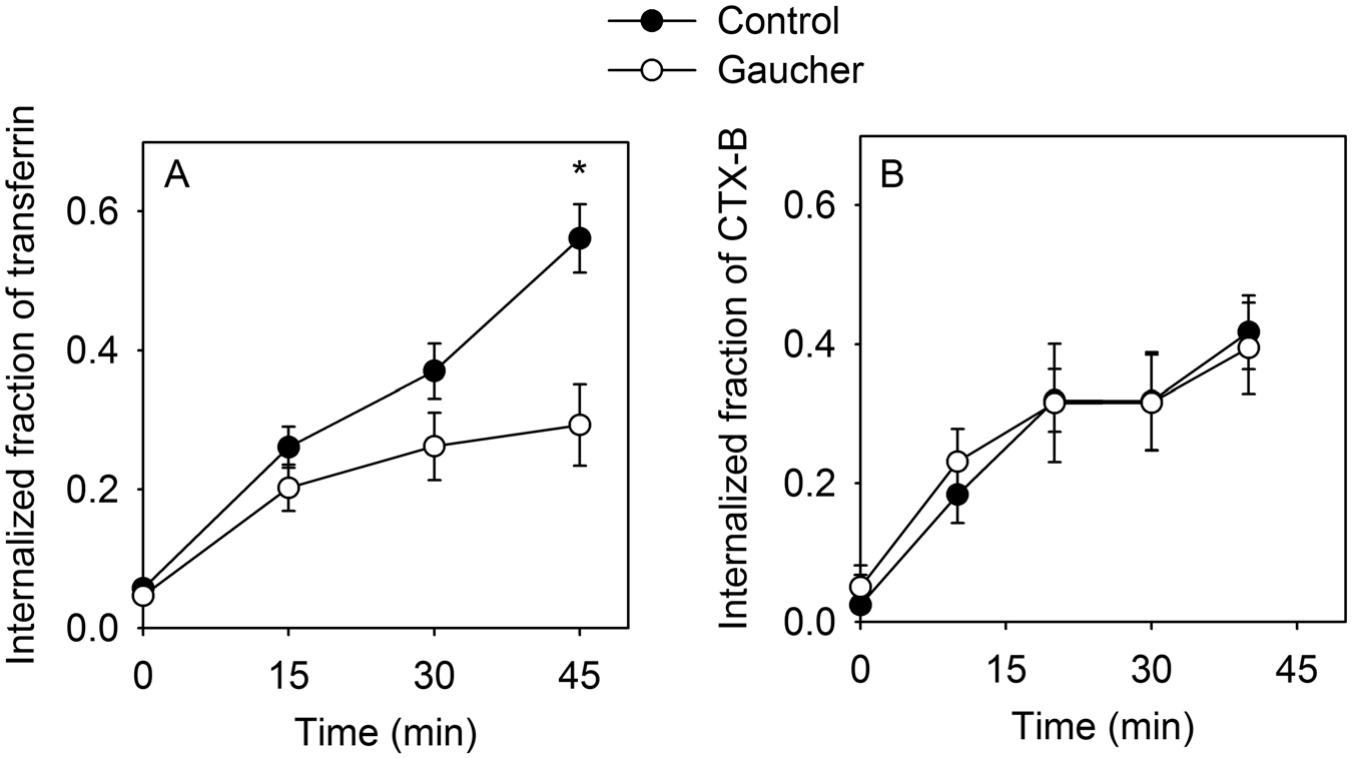
Quantitative evaluation of the endocytosis of transferrin and subunit B of cholera toxin. Cells were incubated with fluorescent transferrin or subunit B of cholera toxin at 37 °C followed by staining with DAPI and a membrane marker (anti-CD14). The cytoplasmic and membrane masks were defined by detecting nuclei and the membrane using the Wählby algorithm and manually-seeded watershed segmentation, respectively. The endocytosed-fraction of the ligands was determined as the relative fraction of fluorescence intensity in the cytoplasmic mask compared to the total cellular fluorescence on a cell-by-cell basis. Error bars indicate the standard error of the mean (n ≈ 100–200 cells from three independent experiments). (*p < 0.05 for the difference between control and Gaucher-type cells at 40 or 45 min).

Since membrane stiffness and viscosity have been linked to endocytosis^28^, the results described in the previous sections led us to assume that the rate of endocytosis of certain ligands may be affected by the Gaucher phenotype. Therefore, we carried out quantitative analysis of endocytosis of transferrin receptor and subunit B of cholera toxin, typical examples for non-raft and raft-dependent internalization pathways, respectively^35,36^. While ∼60% of transferrin was internalized in 45 min by control cells, CBE-treated Gaucher macrophages significantly lagged behind with ∼30% of cell-bound transferrin being present intracellularly (Fig. 5, Fig. S8). Contrary to transferrin, the endocytosis rate of subunit B of cholera toxin was comparable in control and Gaucher macrophages with ∼40% being endocytosed in 40 min. In conclusion, both the FRAP and the endocytosis experiments imply that glycosphingolipid accumulation present in Gaucher macrophages mainly affects the non-raft components of the cell membrane as far as their properties related to lateral diffusion and membrane dynamics are concerned.

### The effect of glycosphingolipid accumulation in the plasma membrane on STAT-mediated signaling

**Figure 6:**
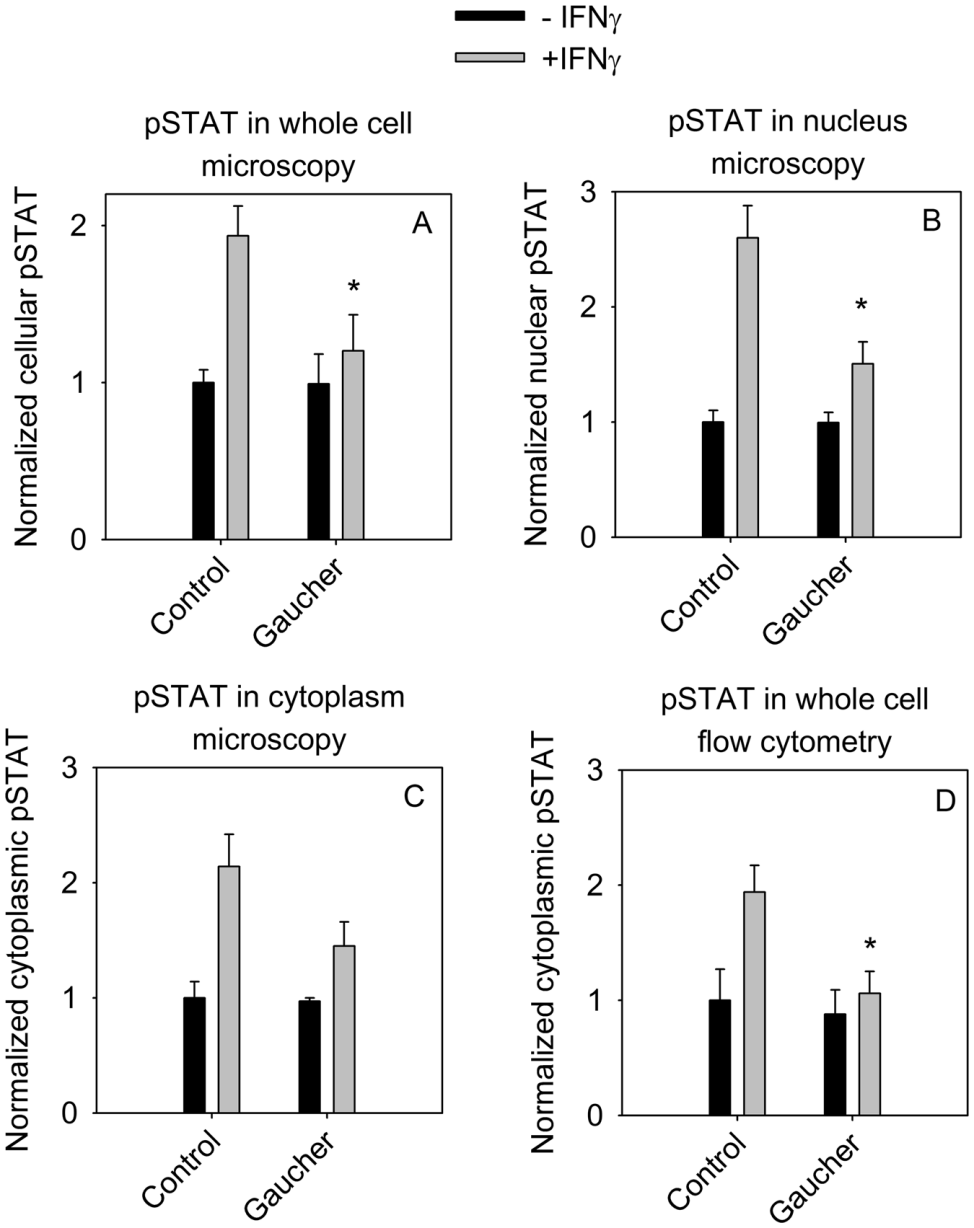
Effect of the Gaucher phenotype on STAT phosphorylation. Control THP-1-derived macrophages and those exhibiting the Gaucher phenotype were serum-starved overnight followed by a 30-min stimulation with 100 ng/ml IFN-γ. Cells were analyzed by microscopy (**A–C**) or flow cytometry after trypsinization (**D**). The level of tyrosine phosphorylated STAT1 was calculated in the whole cell (**A**), in the nucleus (**B**) or in the cytoplasm (**C**) using quantitative image analysis, or it was determined from the mean of flow cytometric histograms (**D**). The fluorescence intensity values were normalized to the unstimulated control. The error bars represent the standard error of the mean of four independent experiments in the case of microscopy and three independent experiments in the case of flow cytometric results. Control and Gaucher-type cells were compared by two-way ANOVA followed by Tukey’s HSD test (*p < 0.05 for the difference between IFNγ-stimulated control and Gaucher cells).

Since the Gaucher phenotype seems to influence the biophysical and cell biological properties of the cell membrane, we aimed at characterizing the difference between control and Gaucher cells regarding signal transduction. Interferon-gamma (IFNγ)-induced tyrosine phosphorylation and nuclear translocation of STAT is an important pathway in macrophages, and STAT1 has been shown to integrate two signals typically initiated by a cytokine and a pathogen-derived molecule, with both of them required for macrophage activation^37^. Control and CBE-treated Gaucher macrophages were serum-starved overnight, then stimulated with IFNγ and subsequently immunostained for total STAT1 and phosphorylated STAT1. Cells were also labeled with fluorescent transferrin and DAPI to segment the confocal images to the cytoplasm and the nucleus (Fig. S9). The Gaucher phenotype did not have any effect on the basal level of STAT phosphorylation, but the extent of IFNγ-induced STAT1 activation in the whole cell and in the nucleus were significantly lower in Gaucher-model cells (Fig. 6, Fig. S9). The Gaucher phenotype induced a nonsignificant decrease in the cytoplasmic level of pSTAT1 (Fig. 6), while it did not have any effect on the level of total STAT1 (quantitative analysis not shown, representative images can be found in Fig. S9). We have independently confirmed the results of these microscopic measurements using flow cytometry. Serum-starved cells were stimulated with IFNγ like in microscopic experiments, followed by trypsinization and staining for total STAT1 and phosphorylated STAT1. While STAT1 expression was not influenced by the Gaucher phenotype or IFNγ stimulation, the IFNγ-induced increase in the tyrosine phosphorylation of STAT1 was significantly attenuated in Gaucher-type cells (Fig. 6, Fig. S10). These results imply that the gross cellular alterations induced by accumulation of glycosphingolipids in the plasma membrane and other membranes lead to inhibition of STAT1 signaling.

## Discussion

The biological properties of the plasma membrane depend on its biophysical characteristics which are, in turn, influenced by the individual and ensemble properties of its lipid and protein constituents^38^. The aim of the current manuscript was to elucidate the effects of glycosphingolipid accumulation on the physical and cell biological properties of the cell membrane in an *in vitro* model of Gaucher disease and to reveal novel molecular mechanisms behind the emergence of the disease symptoms.

Generalized polarization and fluorescence anisotropy measurements revealed that the plasma membrane became less hydrated and more restrictive to rotational diffusion as a result of glycosphingolipid accumulation. The reported 2–10-fold increase in the glycosphingolipid content in *in vitro* and *in vivo* models of Gaucher disease are substantial enough to give rise to such alterations in the biophysical properties of the cell membrane^7–9^. We next performed FRAP experiments to measure the lateral mobility of membrane constituents. FRAP is a robust and model-independent approach, and although the absolute diffusion coefficients provided by this method are sensitive to bleaching correction artifacts, the relative changes are as reliable as those reported by fluorescence correlation spectroscopy (FCS). In addition, FRAP also reveals the immobile fraction which is not detectable by FCS^39^. According to FRAP, the lateral mobility of non-raft protein and lipid constituents was significantly restricted in Gaucher-type cells, while raft-resident proteins and lipids were either unaffected or less significantly inhibited in their lateral mobility. Two-photon microscopy of Laurdan-labeled cell membranes revealed that the fractional area of membrane exhibiting low hydration, corresponding to raft-like domains, increased as a result of glycosphingolipid accumulation. The raft-like phase became so abundant that it formed a continuous, percolating phase isolating and restricting the movement of constituents residing in the non-raft phase explaining why non-raft components were preferentially affected by glycosphingolipid accumulation^40^. When interpreting the results of FRAP experiments it must be kept in mind that diffusion of membrane proteins and lipids in restricted displaying fast local motion within a confined zone and slow hopping between adjacent compartments according to single particle tracking and super-resolved FCS measurements^41^. Diffusion parameters revealed by FRAP are determined by both of the above diffusion processes. Although FRAP is unable to resolve the diffusion coefficient characterizing local motion within a confinement zone, our conclusions are valid since they are based on the assumption that enlargement of glycosphingolipid-enriched domains leads to a lower rate of long-range diffusion due to enhanced confinement of non-raft membrane components. It is noteworthy that highly dissimilar membrane constituents (lipids, GPI-anchored and transmembrane proteins) have been shown to display comparable diffusion characteristics as far as long-range diffusion is concerned suggesting that the barriers restricting diffusion affect all of them in a similar way^42^.

AFM is another sensitive tool for interrogating the biophysical properties of the membrane^30,31^. Step-like decreases in the force measured by the device in the retraction phase correspond to detachment of single membrane tethers from the AFM cantilever. These membrane tethers correspond to nanotubes which, when formed as a result of physiological processes, are responsible for intercellular communication^43^. In-plane membrane tension, bending stiffness and membrane-to-cytoskeleton attachment contribute to the tether force^29^. It has been shown that approximately 50–75% of the force exerted by a single membrane tether on the cantilever is associated with the cytoskeleton and the connection of actin to the membrane^28,29,32,44^. The 10% decrease in tether force in Gaucher-type cells implies that one of the above three components decreased upon glycosphingolipid accumulation in the cell membrane. Although the parameters contributing to tether force also influence cell shape^28^, the 10% decrease in tether force was not sufficient to lead to measurable changes in the shape of cells. The fact that the number of membrane tethers formed was more than two-times higher in Gaucher-type cells than in control ones suggests that the resistance of the membrane to bending was reduced. The formation of membrane tethers or nanotubes involves the generation of negatively and positively curved membrane at the base and around the circumference, respectively. The accumulation of inverted cone-shaped glycosphingolipids in the outer leaflet may promote the formation of highly positively curved membranes and thereby can explain the more frequent formation of membrane tethers. Lipid rafts and glycosphingolipids have already been linked to the formation of membrane nanotubes^45,46^. Since the formation of membrane tethers upon retracting the AFM cantilever does not involve biological processes, our results imply that the altered biophysical properties of the membrane upon glycosphingolipid accumulation per se lead to easier formation of membrane tethers. As membrane nanotubes are involved in immunoregulation, their altered number in Gaucher-type cells may contribute to immune system irregularities in Gaucher disease^47,48^.

Gaucher cells turned out to be more elastic than control ones according to determination of Young’s modulus which is influenced by the properties of the cell membrane and the cytoskeleton. It is unknown whether the dynamics of the cytoskeleton in general, and actin filaments in particular, is directly modified in Gaucher disease. However, it has been shown that alterations in the lipid composition of the cell membrane lead to changes in the elastic properties of the plasma membrane and the cell, and that these changes depend on the actin meshwork^33,49^. It has been hypothesized that anchoring of the actin cytoskeleton to the membrane is behind this phenomenon. Indeed, it has been shown that lipid raft domains are involved in connecting actin to the cell membrane^50,51^. We assume that the increased area of lipid rafts in glycosphingolipid-enriched membranes of Gaucher cells leads to rearrangement or dilution of actin-to-membrane linkages with consequent changes in the dynamics of the actin cytoskeleton.

The aforementioned changes in the biophysical properties of the plasma membrane led to significant alterations in cell biological processes connected to the cell membrane:

(i) Although the raft-dependent endocytosis of cholera toxin was not different in control and Gaucher-type cells, the raft-independent, clathrin-dependent endocytosis of transferrin was significantly inhibited upon glycosphingolipid accumulation^35,36^. Generation of the negatively curved membrane of endocytic vesicles becomes more difficult if inverted cone-shaped glycosphingolipids, favoring the formation of positively curved membranes, accumulate in the extracellular leaflet. Although glycosphingolipids are preferentially present in rafts, their concentration also increases in non-raft domains in Gaucher cells, which may hinder the formation of endocytic vesicles outside rafts^7^. Alternatively, the significantly inhibited lateral diffusion of transferrin receptor, revealed by FRAP measurements, may also lead to the decreased rate of endocytosis of transferrin. In contrast, the raft-dependent endocytosis of cholera toxin did not change upon glycosphingolipid accumulation in the cell membrane, because the biophysical properties of rafts did not change substantially in Gaucher cells according to our FRAP measurements.
(ii) IFNγ-induced STAT1 phosphorylation was significantly reduced in Gaucher cells. This diminished STAT signaling may seem to be somewhat surprising at first sight given that lipid rafts are believed to be crucial in the signal transduction of STAT^52^. However, lipid rafts often play a bipartite role by promoting the activation of membrane receptors and keeping their activation under tight control at the same time^53^. While mild cholesterol extraction induces T cell activation, strong cholesterol depletion inhibits the same process^54,55^. Similarly, although lipid rafts inhibit ligand binding by the epidermal growth factor receptor, they potentiate EGFR-dependent signaling^56,57^. We believe that the increased density of glycosphingolipids in Gaucher cells may inhibit binding of IFNγ or some other steps in the signaling pathway of the cytokine receptor. Reduced activation of STAT upon IFNγ stimulation may also play a role in the mild immunosuppression observed in Gaucher patients^47^.

**Figure 7:**
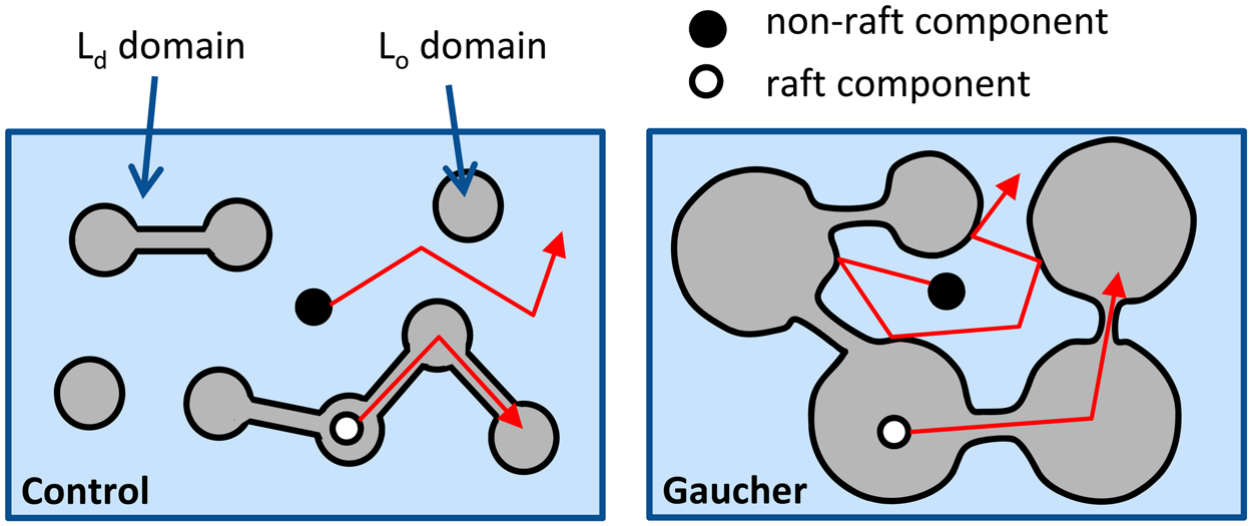
Model for sphingolipid enrichment-induced changes in the plasma membrane. The membrane is assumed to be composed of liquid-disordered (L_d_) and liquid-ordered (L_o_) domains with the latter corresponding to lipid rafts enriched in sphingolipids. Membrane components mainly diffuse within their own domains. Upon sphingolipid enrichment, the physical landscape of the membrane changes due to enlargement of L_o_ domains causing their coalescence and consequent confinement of non-raft components.

In summary, the accumulation of glycosphingolipids in the cell membrane led to the increase in the size of raft-like domains which became a percolating, continuous phase (Fig. 7). According to percolation theory the plasma membrane is an interrupted system in which obstacles form islands in a continuous conducting phase at low obstacle concentrations. However, the obstacles form an obstructed phase isolating islands of the conducting phase from each other at high obstacle concentrations above the percolation transition. The aforementioned model has been put forward several times^58,59^, and the fence-and-picket structure of the plasma membrane also involves a high density of obstacles formed by cytoskeleton-anchored transmembrane proteins^60^. In the context of our experimental system raft-like microdomains correspond to obstacles. An increase in the size of raft-like microdomains and the consequent formation of a continuous raft phase have already been reported upon glucosylceramide loading or changes in temperature^40,61^. While the diffusion of non-raft lipids and proteins was found to be significantly inhibited due to the corralling effect of the percolating raft phase, the movement of raft components was not significantly affected. A similar bipartite effect of glycosphingolipid accumulation was observed for endocytosis. The accumulation of raft domains and inverted cone-shaped lipids in the extracellular leaflet made the membrane more disposed to tether formation and inhibited IFNγ-induced STAT signaling. The results not only reveal new potential cellular pathways behind the symptoms of Gaucher disease, but also characterize the profound changes associated with glycosphingolipid accumulation in the plasma membrane.

## Materials and Methods

### Plasmids, transfection and cell culture

THP-1 cells, obtained from the American Type Culture Collection (ATCC, Manassas, VA), were cultivated according to their requirements in the presence of 10% fetal calf serum (FCS). For serum-starvation, the cells were kept in the above medium containing 0.1% FCS overnight^62^. THP-1 monocytes were differentiated into macrophages with phorbol 12-myristate 13-acetate (PMA; Sigma, P8139) at a final concentration of 5 ng/ml to achieve sufficient differentiation, but to prevent unspecific gene expression changes as much as possible^63^. In order to produce Gaucher macrophage cells *in vitro* THP-1 cells were cultured in the presence of both PMA and conduritol B epoxide (CBE), an irreversible inhibitor of glucocerebrosidase, at a concentration of 500 µM for four days^7,9^. The ErbB2-mYFP plasmid (ErbB2 fused to monomeric YFP) was the kind gift of Donna Arndt-Jovin (Max Planck Institute for Biophysical Chemistry, Göttingen, Germany) and has been described previously^64^. GFP-GPI plasmid was a kind gift from Jennifer Lippincott Schwartz (NIH, Bethesda, MD, USA). The transferrin receptor-GFP (TrfR-GFP) plasmid was generated by Michael Davidson (Florida State University, Tallahassee, FL) and purchased from Addgene (Addgene plasmid #56488). All transfections were carried out by electroporation using Amaxa Nucleofector II (program: V-001; reagent: Lonza Human Monocyte Nucleofector Reagent). According to the suggestion of the manufacturer 5·10^5^ cells were transfected with 2 µg plasmid DNA.

### Fluorescence recovery after photobleaching (FRAP) measurements

For characterizing the mobility of non-raft and raft-specific lipids TR-DHPE (Texas Red dihexadecanoyl–phosphatidylethanolamine, Thermo Fisher Scientific, T1395MP) and BODIPY FL C_5_-Ganglioside GM1 (Thermo Fisher Scientific, B13950), respectively, were used according to their specifications. While TR-DPHE has been shown to partition preferentially into the L_d_ domain^21^, Bodipy FL-labeled GM1 is an indicator of lipid rafts^22^. In order to determine the mobility of non-raft proteins cells were transfected with TrfR-GFP, the archetypical marker of non-raft microdomains^25^, while ErbB2-mYFP and GFP-GPI were used as protein markers loosely and strongly associated with lipid rafts, respectively^23,24^.

FRAP measurements were performed on cells cultured in the presence of PMA or PMA and CBE for four days using an Olympus Fluoview FV1000 confocal microscope. The experiments were carried out at 37 °C using a 60× oil immersion objective (NA = 1.35). TR-DHPE was excited at 543 nm and its emission was collected between 612 and 662 nm. ErbB2-mYFP was excited at 514 nm and its fluorescence was measured between 510 and 610 nm, while TrfR-GFP and GFP-GPI were excited at 488 nm, and their fluorescence was monitored at 527–627 nm. The size of the bleached region was approximately 1 μm. Recovery curves were analyzed with a custom-written Matlab program (Mathworks, Natick, MA; http://peternagy.webs.com/frap). A background region of interest (ROI) was drawn outside cells and the membrane of the bleached cell was determined by performing manually-seeded watershed segmentation in every image so that the membrane mask moved with the changing shape of the membrane (Fig. S3)^65^. Raw intensity values were double-normalized to the pre-bleach intensity and to bleaching taking place during recovery:

1 $$R(t)=\frac{I(t)-bg(t)}{I(0)-bg(0)}\frac{T(0)-bg(0)}{T(t)-bg(t)}$$

where *I*, *T* and *bg* are the mean fluorescence intensities of the bleached ROI, the whole cell membrane and the background, respectively, and *t* and 0 in parentheses refer to the intensity measured at time *t* after the bleaching pulse and before bleaching, respectively. Double-normalized FRAP curves were averaged, and the following empirical equation was fitted to the mean recovery curves, *R*(*t*):

2 $$R(t)=R(0)-a+a(1-b)(1-{e}^{-\frac{t}{\tau }})$$

where *a* is the extent of bleaching, *b* is the immobile fraction and τ is the empirical recovery time constant.

### Determining membrane fluidity and hydration with TMA-DPH and Laurdan

4′-(trimethylammonio)-diphenylhexatriene (TMA-DPH) and Laurdan (6-dodecanoyl-N,N-dimethyl-2-naphthylamine), purchased from Sigma-Aldrich, were dissolved in tetrahydrofuran and dimethyl sulfoxide, respectively. Trypsinized cells were resuspended in Hank’s buffer at a concentration of 10^7^/ml and labeled with 2 μM TMA-DPH or 2.5 μM Laurdan at room temperature for 20 min. After TMA-DPH labeling cells were diluted in Hank’s buffer without washing to a concentration of 10^6^/ml for fluorescence anisotropy measurements, whereas Laurdan-labeled cells were washed once and resuspended at a concentration of 10^6^/ml in Hank’s buffer. Fluorescence measurements were carried out with a Fluorolog-3 spectrofluorimeter (Horiba Jobin Yvon, Edison, NJ). The temperature of the cuvette holder was adjusted to 37 °C by a circulating water bath. TMA-DPH was excited at 352 nm and its emission was measured at 430 nm. The fluorescence anisotropy (r) of TMA-DPH was measured in the L-format according to the following formula^66^:

3 $$r=\frac{{I}_{vv}-G{I}_{vh}}{{I}_{vv}+2G{I}_{vh}}$$

where *I*_vv_ and *I*_vh_ are the vertical and horizontal components, respectively, of the fluorescence excited by vertically polarized light, and G is an instrument-specific correction factor characterizing the different sensitivity of the detection system for vertically and horizontally polarized light. Laurdan was excited at 350 nm and its emission was detected in the blue range of its emission spectrum at 435 nm (*I*_blue_) and at the red edge at 500 nm (*I*_red_). Generalized polarization (GP) of Laurdan fluorescence was calculated according to the following formula^27^:

4 $$GP=\frac{{I}_{blue}-{I}_{red}}{{I}_{blue}+{I}_{red}}$$

### Laurdan two-photon microscopy

Attached cells were labeled with the same concentration of Laurdan as described in the previous section followed by two-photon microscopic measurements at 37 °C. Data were collected with a custom-built microscope built around an Olympus upright frame with a Femto2D scanner (Femtonics Ltd, Budapest, Hungary). Laurdan was excited using an XLUMPlanFL N 20x water immersion objective (NA = 1.0) and a MaiTai pulsed laser (Spectra-Physics, Santa Clara, CA) tuned to 720 nm. The red edge emission of Laurdan (*I*_red_) was detected using a 505LP filter, while its fluorescence in the blue range (*I*_blue_) was recorded between 450 and 470 nm^67^. Images were processed with the DipImage toolbox (Delft University of Technology, Delft, The Netherlands) under Matlab. The generalized polarization was calculated according to equation 4 after background correction of both images followed by applying a color lookup table.

### Measurement of endocytosis

5 × 10^4^ THP-1 cells differentiated to macrophages in the presence or absence of CBE were serum-starved in Ibidi 8-well µ–Slide chambers overnight followed by incubation with AlexaFluor647-transferrin (Thermo Fisher Scientific) at a concentration of 25 µg/ml or with AlexaFluor647-labeled subunit B of cholera toxin (Thermo Fisher Scientific) at a concentration of 50 µg/ml for 0–45 minutes at 37 °C. After incubation, cells were put on ice to prevent further endocytosis followed by fixation with 3.7% formaldehyde. In order to locate and identify cells we adapted a method based on semi-automatic detection of nuclei and the cell membrane^68^. The plasma membrane was labeled with fluorescein-conjugated anti-CD14 (Thermo Fisher Scientific, MHCD14014) and nuclei were stained with DAPI (Sigma-Aldrich) after permeabilization with 0.01% Triton-X-100 followed by fixation with 1% formaldehyde. Images were recorded with a Zeiss LSM880 confocal microscope in three fluorescence channels using a Plan-Apochromat 63× oil immersion objective (NA = 1.43). DAPI-stained nuclei were imaged using excitation at 405 nm and fluorescence detection between 410–483 nm. Fluorescein was excited at 488 nm and its emission was measured between 491–624 nm. The fluorescence of AlexaFluor647 was excited at 633 nm and detected between 638–755 nm. Image analysis was carried out with DipImage and Matlab. Briefly, nuclei were detected using the Wählby algorithm^69^ followed by identification of the cell membrane, labeled by anti-CD14, using the seeded watershed algorithm using nuclei shrunk to single pixels as seeds^65^. The membrane was thickened inwardly and this shape was used as the membrane mask. The area inside the membrane mask was defined as the intracellular mask. Image segmentation is demonstrated in Fig. S8. Images were background-corrected before quantitative evaluation. The fluorescence intensity measured in the AlexaFluor647 channel in the intracellular mask was divided by the total cellular fluorescence (membrane mask + intracellular mask) on a cell-by-cell basis and it was interpreted as the fraction of endocytosed ligand.

### Measurement of membrane elasticity and tether formation by atomic force microscopy

Cells seeded on a borosilicate glass coverslip were placed in a temperature-controlled (37 ± 0.1 °C) closed fluid cell (Bioheater, Asylum Research, Santa Barbara, CA, USA) mounted on the sample stage of an Olympus IX81 inverted microscope and mechanically manipulated in contact mode with an MFP3D atomic force microscope (Asylum Research, Santa Barbara, CA, USA). Force curves were taken with a silicon nitride cantilever (MSCT-AUHW, D lever, nominal resonance frequency and spring constant 15 kHz and 0.03 N/m, respectively (Veeco, Plainview, NY, USA)). The cantilever was calibrated using the thermal method yielding a typical spring constant of 0.03–0.04 N/m^70^. The AFM tip was positioned above a cell and from a distance of about 6–8 μm from its surface the tip was pressed into the cell at 2 μm/s velocity until the load reached 1 nN. Then the tip dwelled on the cell surface for 5 s and was retracted with a velocity of 2 μm/s. At least 10 cells were manipulated from each cell type. Force-volume maps were obtained in 16×16 matrices positioned over individual cells. The retraction phase of the force curves for determining tether force and enumerating membrane tethers was analyzed by a custom-written Matlab application (http://peternagy.webs.com/atomic-force-microscopy) implementing two automatic step detection algorithms followed by visual correction if required^71,72^. In order to determine Young’s elastic modulus, the indentation force traces were fitted with the Hertz model with the specifications of the cantilever and the pyramidal cantilever tips as input parameters^73^.

### Measurement of the expression, phosphorylation and nuclear localization of STAT1

THP-1-derived control and Gaucher-type macrophages were cultured in Ibidi 8-well chambered coverslips for 5 days followed by overnight serum-starvation and stimulation with 1000 U/ml recombinant human IFNγ (R&D Systems, Minneapolis, MN) at 37 °C for 30 min. Afterwards cells were stained with AlexaFluor647-conjugated transferrin at a concentration of 25 µg/ml to label the cell membrane followed by fixation and permeabilization with 3.7% formaldehyde and 0.01% Triton-X-100. Cells were stained with anti-pStat1 (sc-136229, Santa Cruz) mouse monoclonal antibody or with anti-STAT1 (sc-464, Santa Cruz) followed by secondary staining with AlexaFluor546-conjugated goat anti-mouse IgG (Life Technologies, A11030). Nuclei were labeled with 300 nM DAPI followed by fixation with 1% formaldehyde. Microscopic images were taken with a Zeiss LSM880 confocal microscope using a C-Apochromat 40× water immersion objective (NA = 1.2). DAPI was excited at 405 nm and its emission was collected between 410–483 nm. AlexaFluor546 and AlexaFluor647 were excited at 543 nm and 633 nm, respectively, and their fluorescence was measured between 550–630 nm and between 638–755 nm, respectively. Image analysis was carried out with DipImage and Matlab. Nuclear and membrane segmentation was carried out using the DAPI-stained nuclei and transferrin-stained membranes as described for endocytosis measurements. STAT and p-STAT intensities were evaluated in the cell mask corresponding to the area inside the membrane mask including the membrane itself. The nuclear fraction of STAT was calculated by dividing the fluorescence intensity of STAT measured in the nuclear mask by the total cellular intensity.

For flow cytometric analysis, unstimulated and IFNγ-stimulated cells were trypsinized, washed in PBS and stained for total STAT1 and phospho-STAT1 in a way identical to how cells were labeled for microscopy. Nuclear staining with DAPI and membrane staining with AlexaFluor647-transferrin were not carried out with these samples. Cells were measured using a FACSAria III flow cytometer (Becton Dickinson, Franklin Lakes, NJ). AlexaFluor546 was excited with a 561-nm laser line, and its emission was detected between 575–590 nm. Flow cytometric data files were evaluated using FCS Express (DeNovo Software, Glendale, CA).

## Electronic supplementary material

Supplementary information

Supplementary movie S1
