# Supplementary material for: Alterations in the properties of the cell membrane due to glycosphingolipid accumulation in a model of Gaucher disease
Source: Sci Rep. 2018 Jan 9;8:157. doi: 10.1038/s41598-017-18405-8 (PMC5760709; doi:10.1038/s41598-017-18405-8)
Supplement: Supplementary file 1 — Supplementary information [file 41598_2017_18405_MOESM1_ESM.pdf]

Supplementary material to

Alterations in the properties of the cell membrane due to glycosphingolipid accumulation in a model of Gaucher disease

Gyula Batta, Lilla Soltész, Tamás Kovács, Tamás Bozó, Zoltán Mészár, Miklós Kellermayer, János Szöllősi, Peter Nagy

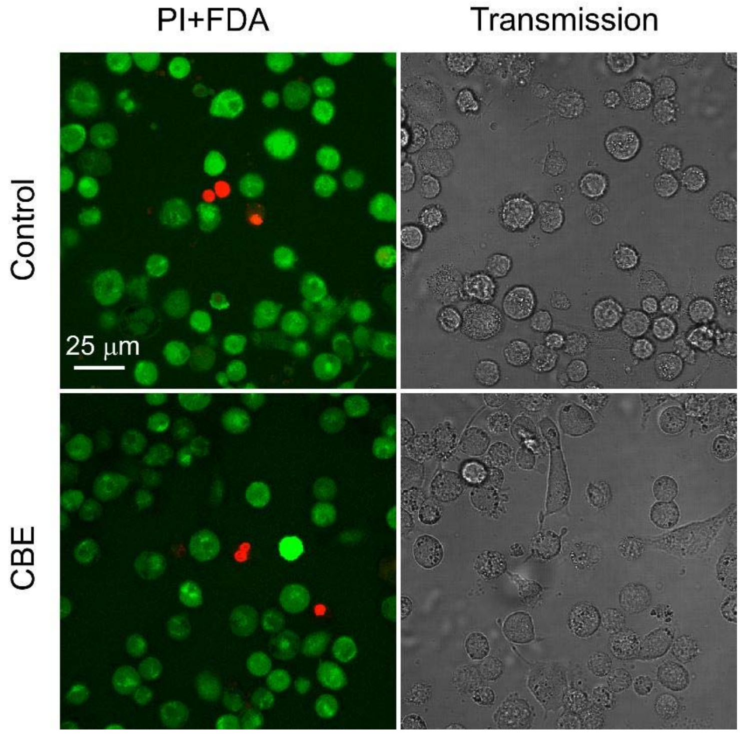

Figure S1. Cell viability is not affected by CBE treatment. Control samples and cells treated with CBE for four days were stained with a mixture of propidium iodide (PI, red channel) and fluorescein-diacetate (FDA, green channel) labeling dead and viable cells, respectively. Staining was performed with 200 μg/ml PI and 25 μg/ml FDA for 15 minutes. Fluorescence overlay images and the corresponding transmission images for control and CBE-treated cells are shown in the figure. The fraction of PI^+ dead cells was 94±1% and 96±1% for control and CBE-treated cells, respectively, based upon analysis of 10 images for both conditions.

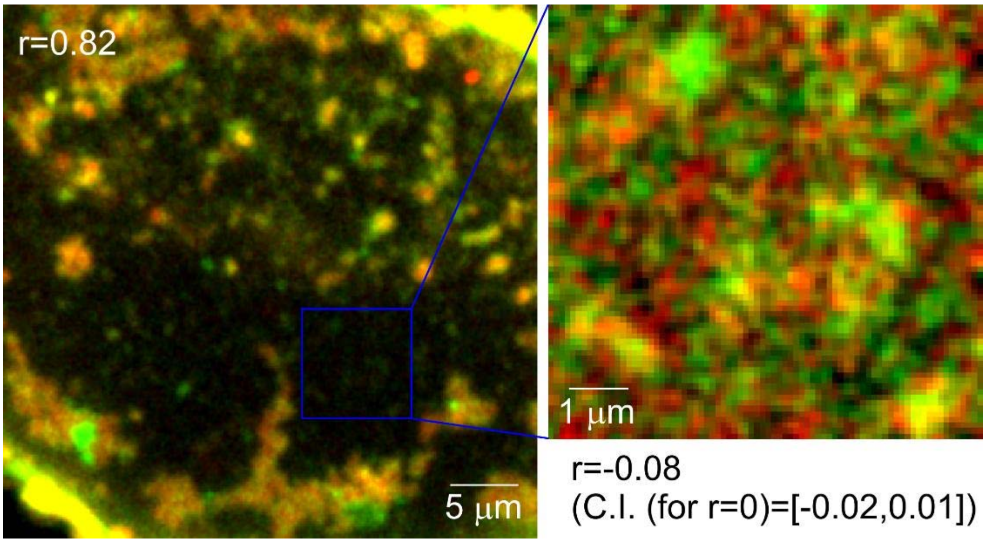

Figure S2. Demonstration of labeling of different membrane domains by TR-DHPE and BodipyFL-GM1. Control cells were labeled with TR-DHPE and BodipyFL-GM1 lipid analogues and a confocal microscopic section of the flat membrane surface adjacent to the coverslip was recorded. The fluorescence of BodipyFL-GM1 and TR-DHPE are shown in the green and red channels, respectively. Although the displayed images were contrast-stretched, the analysis was performed on the original data. Pearson's correlation coefficient was calculated for the whole image (r=0.82) and for a smaller part as well (r= –0.08). The confidence interval (C.I.) for the lack of correlation (r=0) was calculated according to the method of Costes[1]. The bright areas in the image on the left likely correspond to membrane folds often misinterpreted as areas exhibiting colocalization[2].

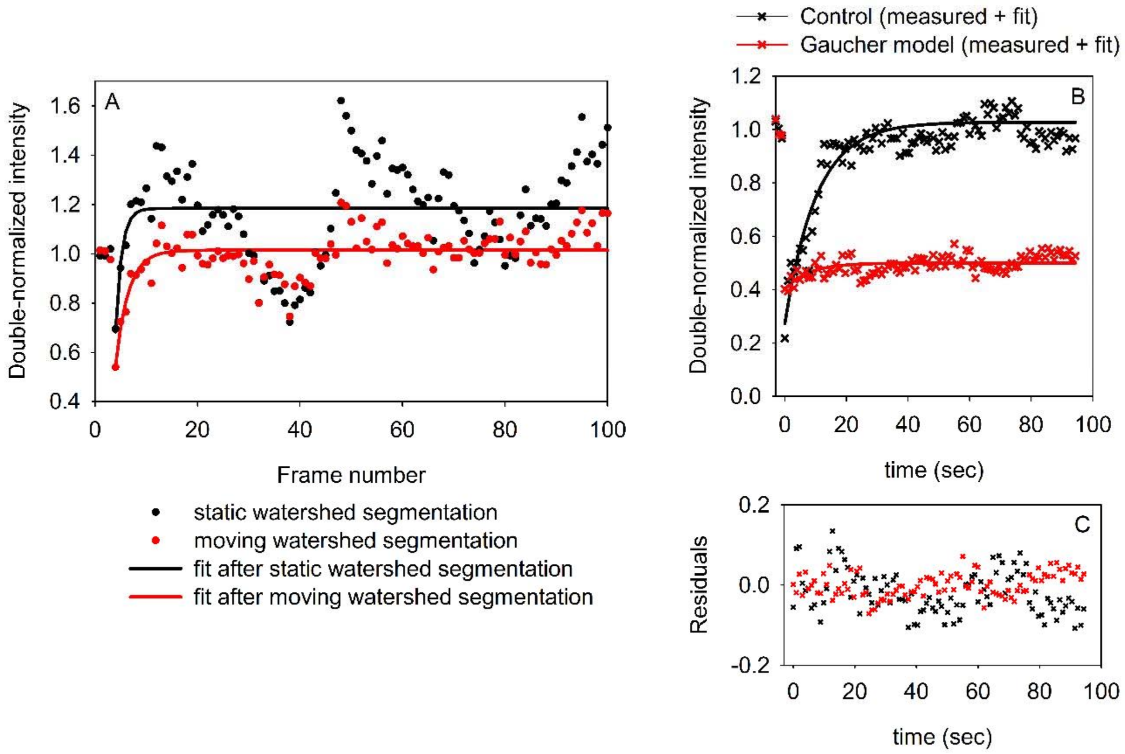

Figure S3. Analysis of fluorescence recovery after photobleaching (FRAP) experiments. Control and Gaucher-type macrophages were labeled with TR-DHPE followed by FRAP analysis. **A.** Due to movement of the cell during the recovery period, a single membrane mask ("static watershed") was not sufficient to produce reliable recovery curves. When the membrane mask was defined in each slice using watershed segmentation ("moving watershed"), movement artifacts were removed. **B-C.** Representative recovery curves measured in control and Gaucher-type macrophages (B) and the residuals from the fitted model (C).

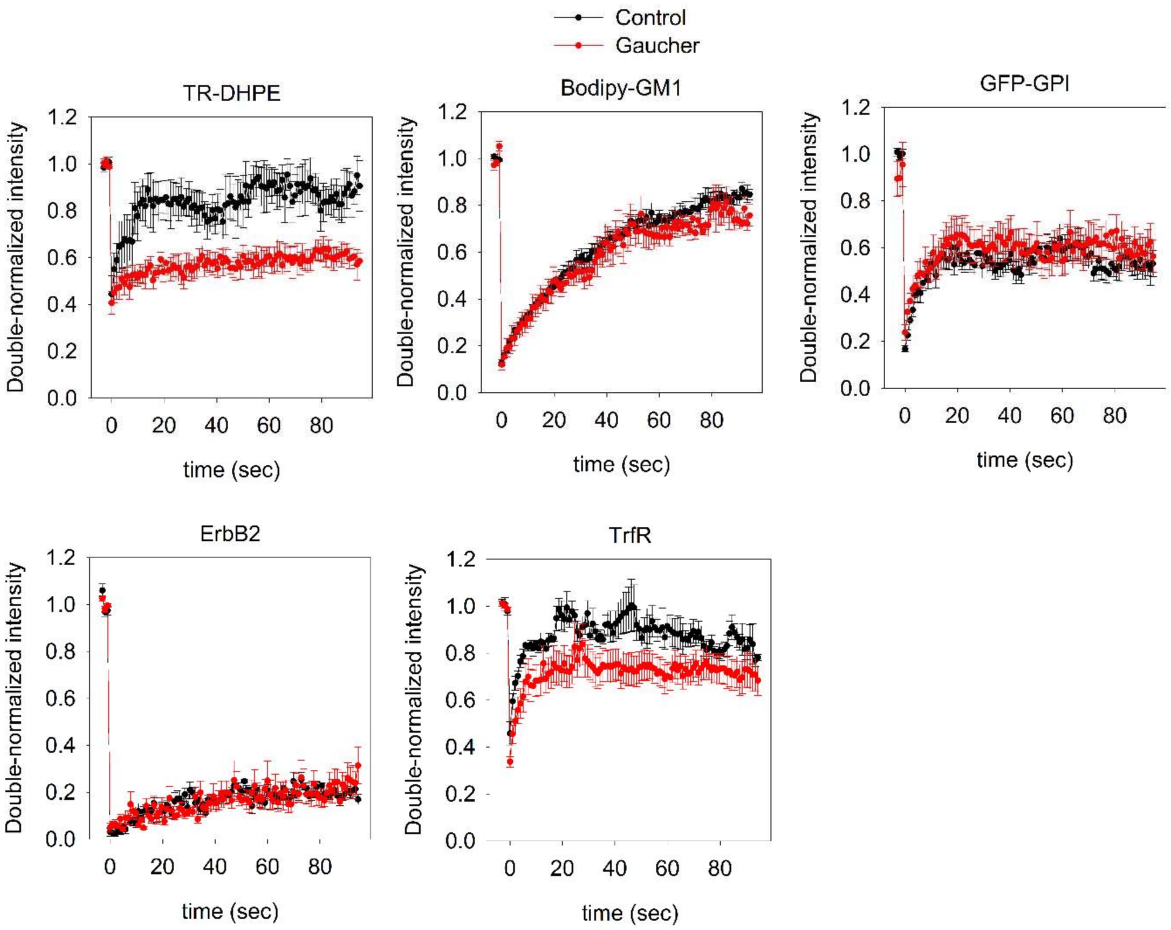

Figure S4. Averaged FRAP curves in control and Gaucher-type cells. Individual fluorescence recovery curves were double-normalized as described in Materials and Methods followed by calculating their mean (±standard error of the mean). The curves represent 10 individual cells from three independent measurements.

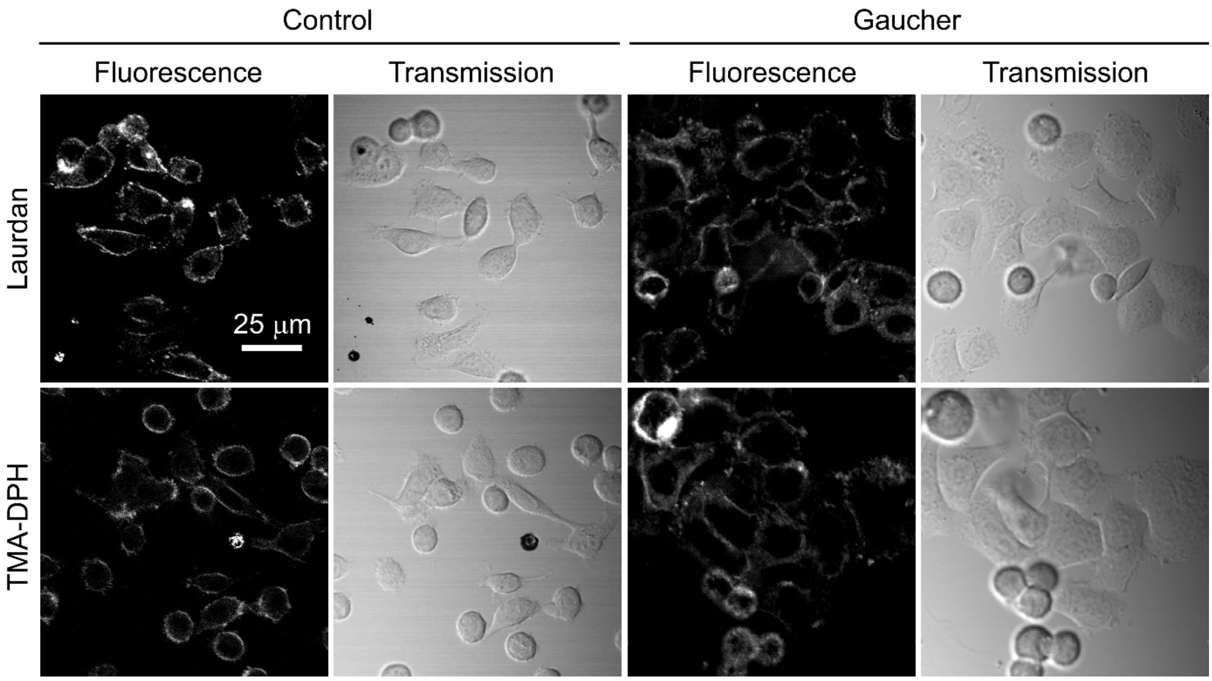

Figure S5. Demonstration of membrane staining with Laurdan and TMA-DPH. Control and Gaucher-type cells were stained with the two indicators as described in Materials and methods, and the fluorescence of Laurdan and TMA-DPH was measured by two-photon microscopy. The images demonstrate preferential staining of the plasma membrane under the applied staining and imaging conditions.

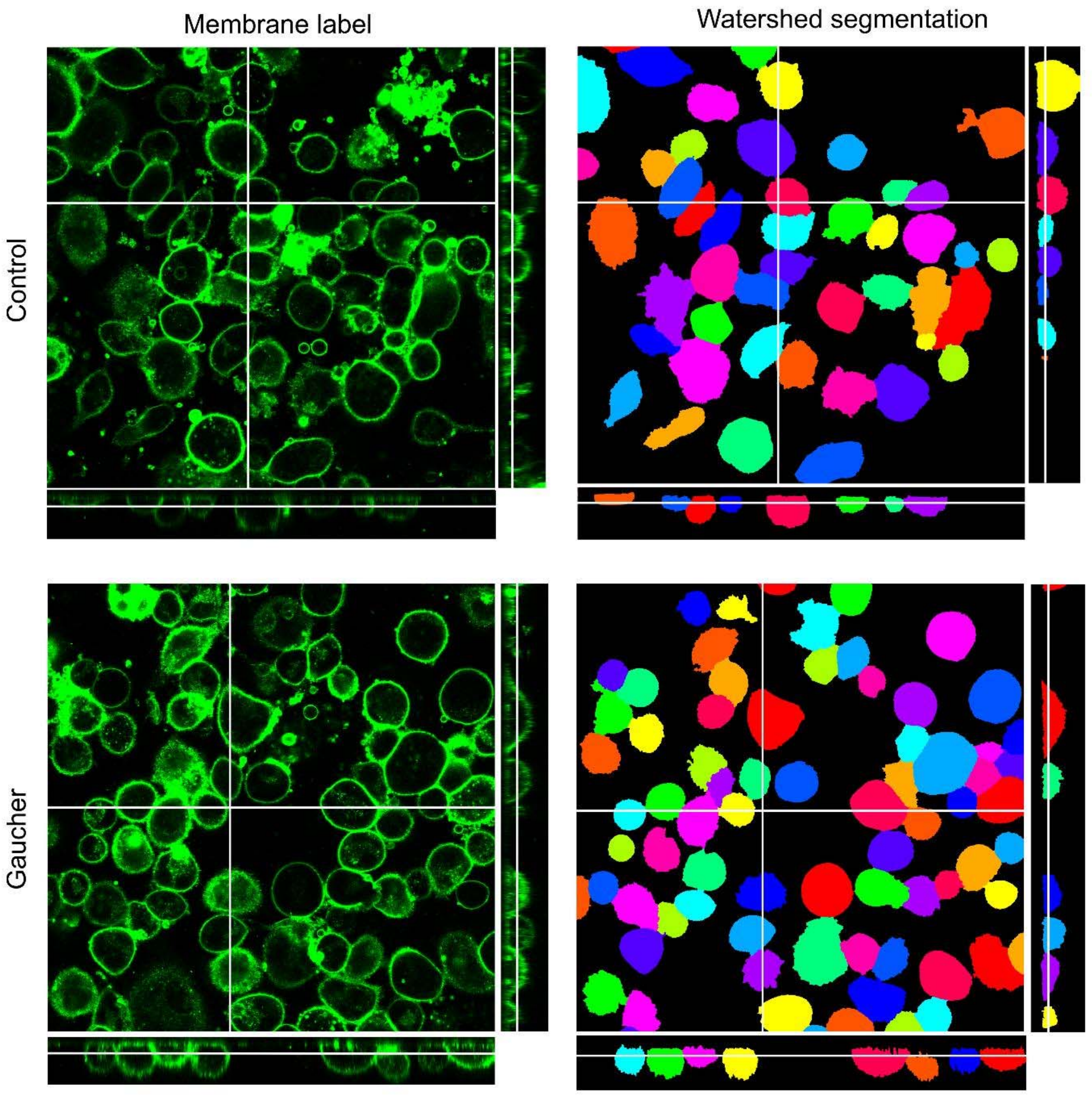

Figure S6. Determination of the relative height of cells. Control and Gaucher cells were labeled with Bodipy FL-GM1 followed by recording confocal stacks using identical microscope settings. Orthogonal views of representative examples are shown on the left. Manually-seeded, three dimensional watershed segmentation was used to identify cells. The color-coded cell masks are shown on the right. The area of the cell, defined as the number of pixels constituting the cell mask in the layer adjacent to the coverslip, and the height, defined as the distance in pixels between the bottom of the cell and its highest pixel in the mask, were calculated. The relative height of individual cells was calculated as the height divided by the area. These normalized cell heights did not differ significantly between the control and Gaucher-type cells (mean $\pm$ SEM was $1.1 \cdot 10^{-3} \pm 2 \cdot 10^{-4}$ and $1.5 \cdot 10^{-3} \pm 3 \cdot 10^{-4}$ for control and Gaucher cells, respectively. n=200 for both samples).

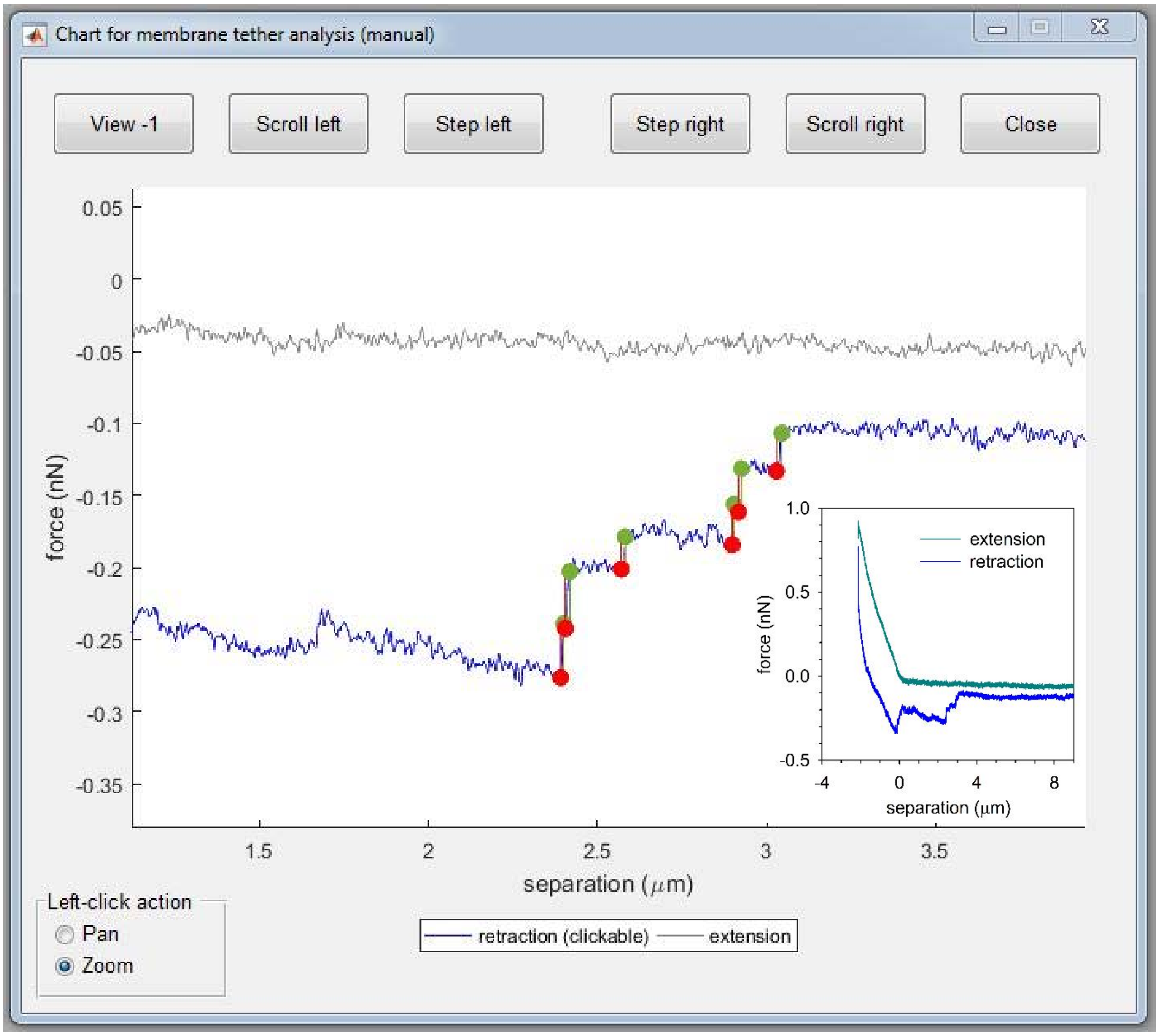

Figure S7. Analysis of membrane tether formation by atomic force microscopy. Membrane tether formation was analyzed in the retraction curves measured by atomic force microscopy in a custom-written Matlab program whose graphical user interface is shown in the figure. The force-separation plot of a full approach-retraction cycle is displayed in the insert in the lower right corner. Automatic detection of step-like changes in the force-separation plot was followed by visual correction. The red and green dots and lines label the detected drops in the force experienced by the AFM cantilever corresponding to severing of single membrane tethers.

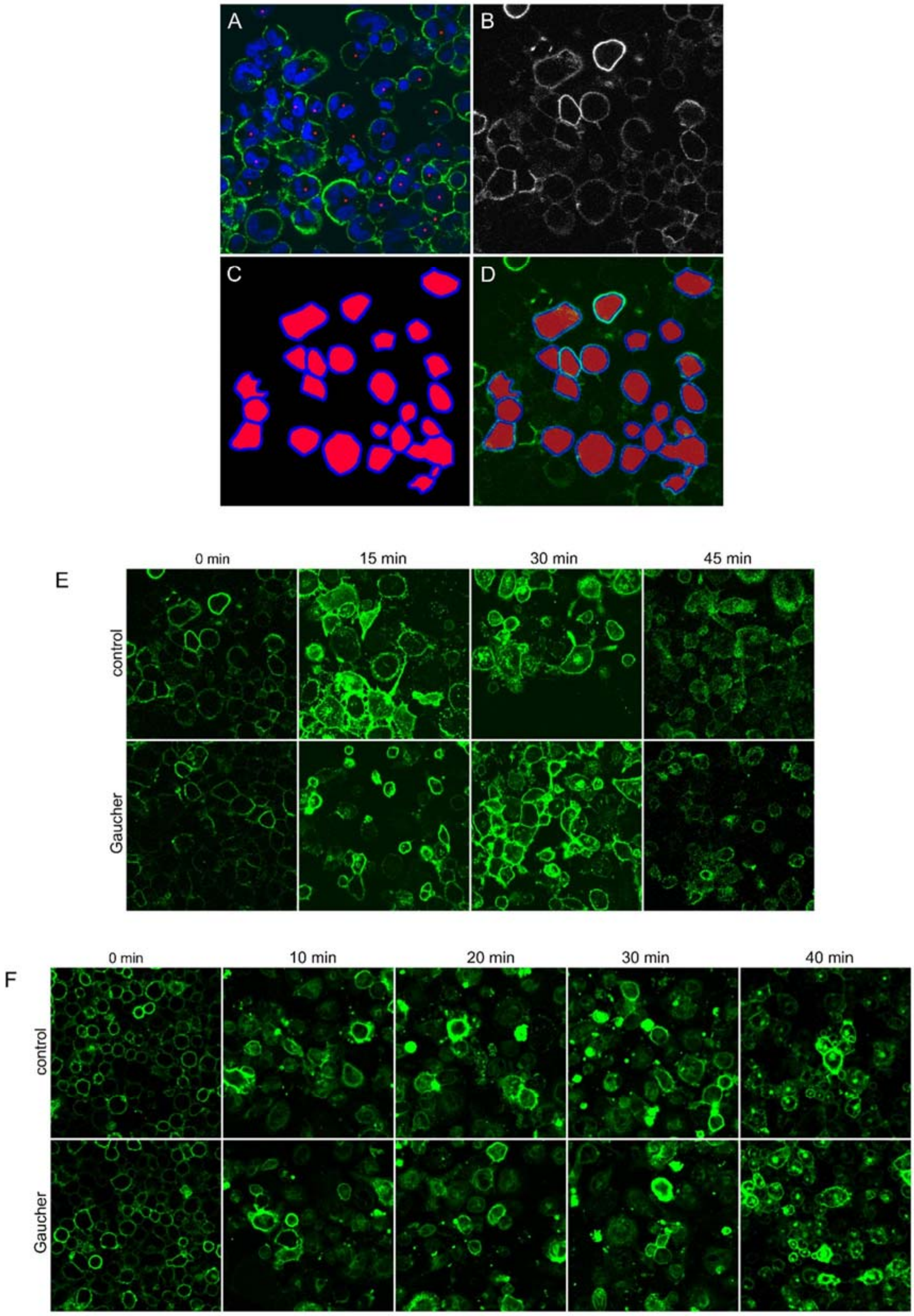

Figure S8. Quantitative evaluation of the endocytosis of transferrin and subunit B of cholera toxin (CTX-B). **A.** Membrane staining with anti-CD14 (green channel) and nuclear staining with DAPI (blue channel) were used for image segmentation. Red rectangles label each identified

cell. **B.** The fluorescence intensity of AlexaFluor647-transferrin is displayed in gray-scale. **C.** The membrane mask (blue) and the intracellular mask (red) identified by nuclear segmentation according to Wählby[3] and manually-seeded watershed segmentation[4] are shown in the image. **D.** The mask shown in C was overlaid on the fluorescence image of AlexaFluor647-transferrin shown in green. **E-F.** Representative images showing the endocytosis of transferrin (E) and CTX-B (F) in control and Gaucher-type macrophages.

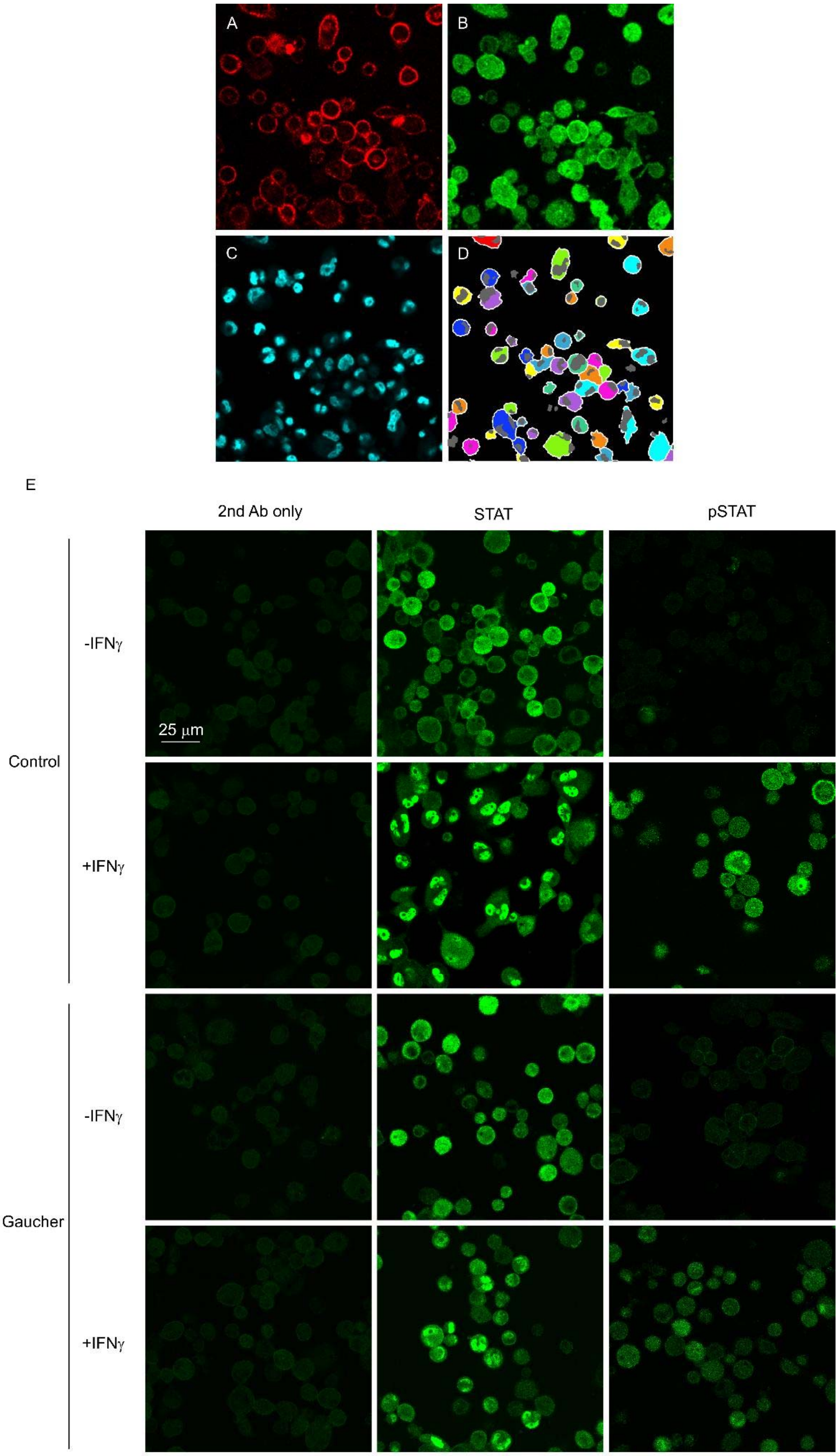
A
B
C
D
E
2nd Ab only
STAT
pSTAT
Control
-IFNγ
+IFNγ
Gaucher
-IFNγ
+IFNγ
25 μm

Figure S9. Measurement of STAT phosphorylation in control and Gaucher-type cells (on previous page).

A-D. Segmentation of confocal images for analyzing STAT phosphorylation. Cells were labeled with AlexaFluor647-transferrin (A), a monoclonal antibody against pSTAT1 (B) and DAPI (C). Nuclei were identified by the Wählby algorithm[3] followed by watershed segmentation using the nuclei as seeds to identify the cell membrane[4]. Segmentation results are shown in D. The white lines and the gray areas correspond to the cell membrane and the nucleus, respectively. Cells with nuclei whose size was below a threshold and nuclei without cytoplasm around them were not evaluated.

E. Representative images for the measurement of tyrosine phosphorylation of STAT1. Control and Gaucher-type cells were starved overnight followed by stimulation with 1000 U/ml recombinant human IFNγ at 37°C for 30 min and staining for total-STAT1 and tyrosine-phosphorylated STAT1. Control samples stained with secondary antibody only, and cells stained with primary antibodies against STAT1 or tyrosine-phosphorylated STAT1 followed by secondary staining are shown in the figure. Images were contrast-stretched in an identical manner to preserve the relative differences between samples.

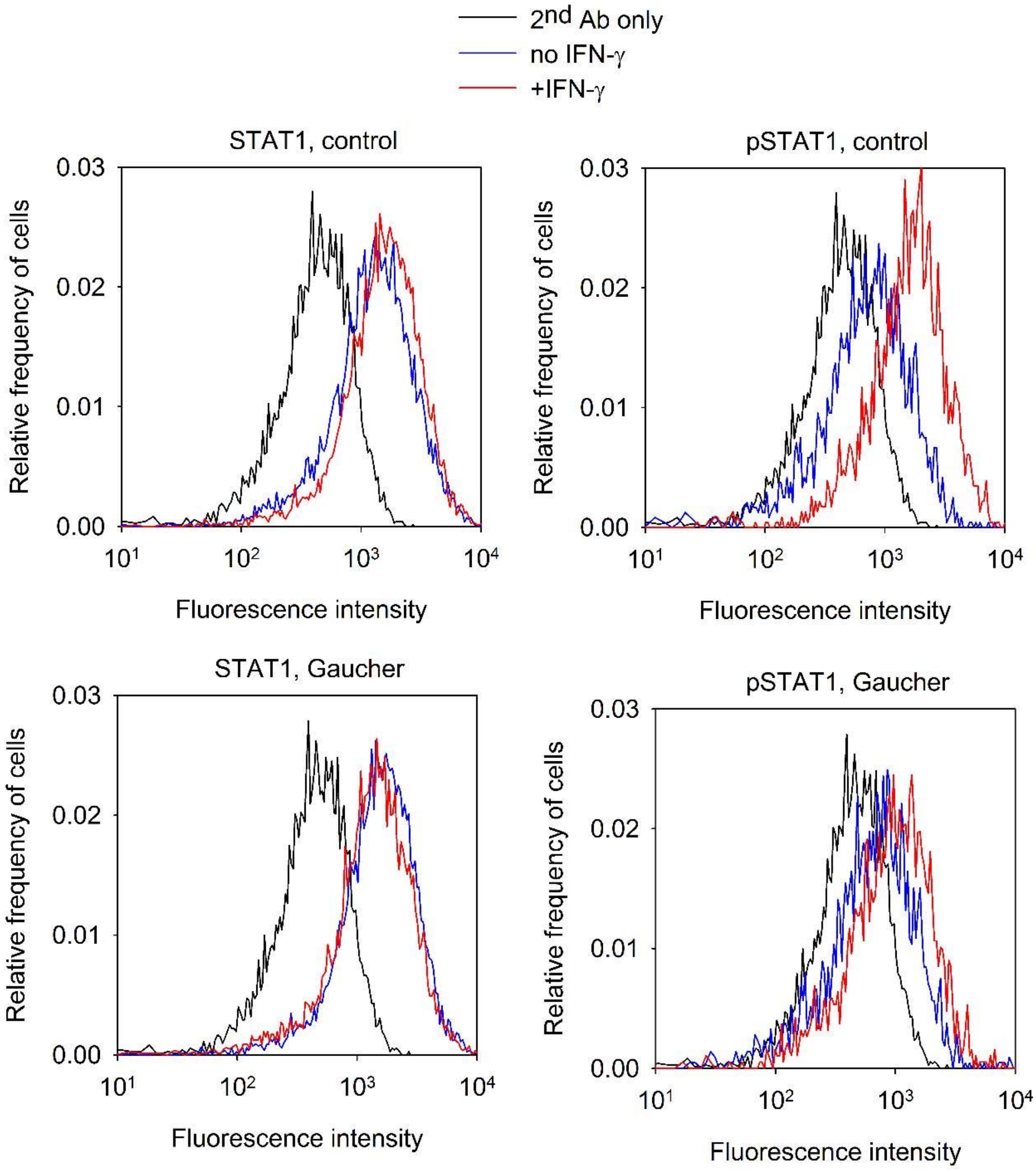

Figure S10. Flow cytometric investigation of the expression and tyrosine phosphorylation of STAT1 in control and Gaucher-type cells. Serum-starved control (top row) and Gaucher-type cells (bottom row) were stimulated with IFN-γ followed by trypsinization and secondary staining against STAT1 or tyrosine phosphorylated STAT1 (pSTAT1). Representative flow cytometric histograms are shown in the figure.

Supplementary references

1 Costes, S. V. *et al.* Automatic and quantitative measurement of protein-protein colocalization in live cells. *Biophys J* **86**, 3993-4003, doi:10.1529/biophysj.103.038422 S0006-3495(04)74439-2 [pii] (2004).

2 Parmryd, I. & Onfelt, B. Consequences of membrane topography. *FEBS J* **280**, 2775-2784, doi:10.1111/febs.12209 (2013).

3 Wählby, C., Sintorn, I. M., Erlandsson, F., Borgefors, G. & Bengtsson, E. Combining intensity, edge and shape information for 2D and 3D segmentation of cell nuclei in tissue sections. *J Microsc* **215**, 67-76, doi:10.1111/j.0022-2720.2004.01338.x (2004).

4 Gonzalez, R. C., Woods, R. E. & Eddins, S. L. in *Digital Image Processing Using Matlab* (eds R.C. Gonzalez, R.E. Woods, & S.L. Eddins) Ch. 10.5, 417-425 (Pearson Prentice Hall, 2004).
